# Omega‐3 polyunsaturated fatty acid supplementation attenuates blood pressure increase at onset of isometric handgrip exercise in healthy young and older humans

**DOI:** 10.14814/phy2.12875

**Published:** 2016-07-20

**Authors:** Christine M. Clark, Kevin D. Monahan, Rachel C. Drew

**Affiliations:** ^1^ Penn State College of MedicineMilton S. Hershey Medical CenterHersheyPennsylvania; ^2^ Penn State Heart and Vascular InstitutePenn State College of MedicineMilton S. Hershey Medical CenterHersheyPennsylvania

**Keywords:** Aging, blood pressure, fish oil, handgrip exercise, heart rate

## Abstract

Aging is associated with alterations of autonomic nerve activity, and dietary intake of omega‐3 polyunsaturated fatty acids, such as eicosapentaenoic acid (EPA) and docosahexaenoic acid (DHA) found in fish oil (FO), can modulate autonomic nerve activity. However, the effect of omega‐3 polyunsaturated fatty acid consumption on age‐related cardiovascular responses at the onset of isometric handgrip exercise, a time of rapid autonomic adjustments, is unknown. Accordingly, 14 young (25 ± 1 years; mean ± SE) and 15 older (64 ± 2 years) healthy subjects ingested 4 g FO daily for 12 weeks. On pre‐ and postintervention visits, participants performed 15‐sec bouts of isometric handgrip at 10%, 30%, 50%, and 70% maximal voluntary contraction (MVC) while beat‐to‐beat systolic, diastolic, and mean arterial blood pressure (SBP, DBP, MAP; Finometer) and heart rate (HR; electrocardiogram) were recorded. All baseline cardiovascular variables were similar between groups and visits, except DBP was higher in older subjects (*P* < 0.05). FO increased erythrocyte EPA and DHA content in both groups (*P* < 0.05). FO attenuated MAP and DBP increases in response to handgrip in both age groups (change from baseline during 70% MVC handgrip pre‐ and post‐FO: young MAPΔ 14 ± 2 mmHg versus 10 ± 2 mmHg, older MAPΔ 14 ± 3 mmHg versus 11 ± 2 mmHg; young DBPΔ 12 ± 1 mmHg versus 7 ± 2 mmHg, older DBPΔ 12 ± 1 mmHg versus 7 ± 1 mmHg; *P* < 0.05). FO augmented the PP (SBP‐DBP) increase with 70% MVC handgrip in both groups (*P* < 0.05), but did not alter SBP or HR increases with handgrip. These findings suggest that FO supplementation attenuates MAP and DBP increases at the onset of isometric handgrip exercise in healthy young and older humans.

## Introduction

Aging is associated with alterations of the autonomic nervous system, with a progressive increase in sympathetic activity (Sundlöf and Wallin 1978; Esler et al. 1995), and a gradual decline in parasympathetic activity (Stratton et al. 2003; Kaye and Esler 2008). Both heightened sympathetic and reduced parasympathetic nerve activity have been linked with increased mortality and development of cardiovascular disease (Hasking et al. 1986; La Rovere et al. 1988, 1998; Meredith et al. 1991). Consequently, it is important to further elucidate the effect of aging on cardiovascular responses to physiological stress that acutely alters autonomic activity to better understand the influence of advancing age on cardiovascular risk.

At the onset of exercise, a physiological stressor, the cardiovascular system undergoes several rapid reflex adjustments, which are mediated primarily by the sympathetic nervous system, as well as the parasympathetic nervous system. These reflex responses at the onset of exercise are primarily a result of feedback due to muscle mechanoreflex activation, and feedforward via central command, with minimal contribution from muscle metaboreflex activation, and are regulated by the arterial baroreflex (Raven et al. 2006). Group III and IV muscle afferent nerve fibers located in skeletal muscle deliver mechanical and metabolic feedback to the medulla oblongata via the dorsal horn of the spinal cord (Kaufman et al. 1983), and this feedback is integrated in the nucleus tractus solitarii (NTS) with afferent input from baroreceptors (Potts 2002). Consequent changes in autonomic outflow from the brain result in reflexive increases in heart rate (HR) and mean arterial blood pressure (MAP), as well as other adjustments, at the onset of exercise, as well as throughout the duration of the exercise (Potts 2002). Previous research has shown that the degree of the HR increase in response to isometric handgrip exercise varies based on age, with older people exhibiting a smaller increase in HR during handgrip (Taylor et al. 1991; Ng et al. 1994; Houssiere et al. 2006; Muller et al. 2012). At the onset of isometric handgrip exercise at a moderate‐to‐high intensity, HR and cardiac output increase less in older compared to younger individuals (Momen et al. 2004; Lalande et al. 2014). In contrast, the MAP increase in response to the onset of isometric handgrip exercise is preserved in older individuals, possibly due to increased arterial stiffness in this population (Momen et al. 2004; Lalande et al. 2014). However, this functional rise in MAP also increases cardiac work, due to greater afterload, and may strain an aged heart that already exhibits blunted responses to autonomic adjustments. As older people have elevated cardiovascular risk, there is a need for identifying interventions that can reduce the cardiovascular response to physiological stress in this population, particularly as brief physiological stress occurs frequently in the activities of daily living.

Dietary intake of omega‐3 polyunsaturated fatty acids, such as eicosapentaenoic acid (EPA) and docosahexaenoic acid (DHA) found in fish oil (FO), has been associated with a reduction in cardiovascular mortality (1999; Sala‐Vila et al. 2016). We have recently shown that FO supplementation decreases central arterial stiffness in older but not young healthy subjects (Monahan et al. 2015). An age‐related increase in arterial stiffness is linked with elevated cardiovascular risk (Seals 2014), so this finding suggests one of the possible mechanisms involved in the cardioprotective effect of FO consumption. Another potential mechanism by which intake of omega‐3 polyunsaturated fatty acids might confer cardiovascular benefit is through modulating autonomic nervous system activity. FO consumption has been shown to reduce resting blood pressure, particularly in older and hypertensive subjects (Geleijnse et al. 2002), and resting HR, especially in individuals with a higher resting HR (Hamazaki et al. 2005; Mozaffarian et al. 2005b; Carter et al. 2012). Consumption of baked or broiled fish by older individuals can lower risk of developing congestive heart failure, a condition associated with increased sympathetic activation (Mozaffarian et al. 2005a). However, the precise effects of omega‐3 polyunsaturated fatty acid consumption on cardiovascular responses to physiological stress are currently unclear. Studies have reported that FO consumption attenuates the HR and total muscle sympathetic nerve activity increases, but not the MAP increase, in response to mental stress (Carter et al. 2013), yet muscle sympathetic nerve activity responses to fatiguing ischemic handgrip and a cold pressor test are augmented, but MAP and HR responses are unchanged (Monahan et al. 2004). Given these contrasting findings and the importance of understanding the effects of omega‐3 polyunsaturated fatty acid consumption on cardiovascular responses to acute physiological stress, further study in this area is warranted.

Accordingly, we examined this concept by measuring cardiovascular responses at the onset of isometric handgrip exercise, a time of rapid reflex neural adjustments, in healthy young and older humans before and after 12 weeks of FO supplementation. As older individuals exhibit a blunted HR but preserved MAP increase at the onset of isometric handgrip exercise (Lalande et al. 2014), and FO supplementation can reduce central arterial stiffness in older people (Monahan et al. 2015), we hypothesized that older subjects would exhibit an attenuated MAP increase at the onset of isometric handgrip exercise following 12 weeks of FO supplementation. Findings from this investigation would provide new clinically relevant insight into the effect of omega‐3 polyunsaturated fatty acid consumption on age‐related cardiovascular responses to acute physiological stress.

## Methods

### Ethical approval

The experimental protocol was approved by the Institutional Review Board at the Penn State Milton S. Hershey Medical Center and was in compliance with the Declaration of Helsinki standards. The purpose of this study and risks of involvement were explained to the subjects, and signed informed consent was obtained.

### Subjects

Fourteen young (6 men and 8 women; age 25.1 ± 0.5 years; height 1.76 ± 0.02 m; weight 77 ± 3 kg) and 15 older (7 men and 8 women; age 63.5 ± 1.7 years; height 1.71 ± 0.03 m; weight 75 ± 4 kg) healthy subjects participated in this study. Subjects were required to meet the following inclusion criteria: good health, normotensive, nonsmokers, no history of cardiovascular disease, and not taking any medications that could affect autonomic or cardiovascular function. Subjects were instructed to refrain from strenuous exercise, caffeine, and alcohol for 24 h and food for 8 h prior to their study visits.

### Intervention

**Figure 1:**
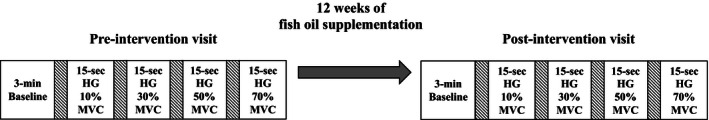
Schematic depiction of the experimental protocol and timeline of the intervention. Shaded area denotes ~1‐min period of rest. HG, handgrip; MVC, maximum voluntary contraction.

Subjects underwent pre‐ and postintervention visits in relation to their FO treatment (Fig. 1). Following completion of the procedures and measurements in the preintervention visit, subjects then began the intervention of ingesting 4 g of highly purified and concentrated FO supplements (omega‐3 acid ethyl esters; Lovaza; GlaxoSmithKline, London, UK) daily for 12 weeks. FO supplementation consisted of 4 × 1 g capsules, each containing at least 900 mg of EPA and DHA, the active ingredients. Pill diaries, pill counts, and phone calls from investigators were employed to assess compliance, and erythrocyte EPA and DHA levels were quantified in a subset of subjects (refer to *EPA and DHA quantification*). Following this period of FO intake, subjects returned for a postintervention visit during which they underwent the same procedures and measurements as in the preintervention visit.

### Experimental protocol

On pre‐ and postintervention visits, a blood sample was taken at the beginning of the visits for EPA and DHA quantification. Subjects’ maximum voluntary contraction (MVC) of the right arm was determined using a handgrip dynamometer (Stoelting Company, Wood Dale, IL). The subject's MVC was used to calculate values for the graded handgrip intensities (10%, 30%, 50%, and 70% MVC), which were then displayed on a screen. During each visit, MAP, systolic (SBP) and diastolic (DBP) blood pressures, and HR were measured at rest during a 3‐min baseline and during 15‐sec bouts of isometric handgrip at 10%, 30%, 50%, and 70% MVC. Subjects had a ~1‐min period of rest between bouts.

### Cardiovascular measurements

Heart rate was measured with a three‐lead electrocardiogram (ECG, Cardiocap/5; GE Healthcare, Waukesha, WI). Beat‐to‐beat MAP, SBP, and DBP were measured with a photoplethysmographic finger cuff (Finometer; FMS, Arnhem, Netherlands). A semi‐automated upper arm cuff (Dinamap; GE Medical System, Milwaukee, WI) was used to obtain three baseline blood pressure measurements, which were used to calibrate the baseline finger cuff signal in offline analysis. MAP, SBP, DBP, and HR were measured continuously at baseline and during handgrip.

### EPA and DHA quantification

The amount of EPA and DHA present in erythrocytes was quantified in a subset of young (*n* = 9) and older (*n* = 8) subjects before and after FO supplementation according to established laboratory methodologies (Harris et al. 2012).

### Data and statistical analyses

An analog‐to‐digital converter sampled data at 400 Hz, and data were displayed and recorded for offline analysis (MacLab 8e; AD Instruments; Castle Hill, NSW). Raw data files were analyzed to produce beat‐to‐beat values for MAP, SBP, DBP, and HR. Pulse pressure (PP) was calculated as SBP minus DBP. Absolute and relative (change from baseline) mean MAP, SBP, DBP, PP, and HR values were calculated for the 3‐min baseline and each of the four 15‐sec bouts of handgrip for the young and older groups during both the pre‐ and postintervention visits. The data are presented as mean ± SEM.

A repeated measures analysis of variance (ANOVA) involving a one within‐ (condition: pre‐ vs. postintervention) and one between‐ (age: young vs. old) factor was used to assess baseline differences in subject characteristics. A two within‐ (condition: pre‐ vs. postintervention, and handgrip intensity: 10%, 30%, 50%, and 70% MVC) and one between‐ (age: young vs. old) factor, repeated measures ANOVA was used to assess differences in cardiovascular responses to handgrip. Post hoc analysis, entailing pairwise comparisons with a Holm–Bonferroni correction, was conducted when significant interactions between factors were identified. Statistical significance was established as *P* < 0.05, and all statistical analyses were performed using SPSS (IBM, Armonk, NY).

## Results

### Subject characteristics at rest

Baseline cardiovascular, and EPA and DHA content, values of the young and older groups are shown in Table 1. Resting MAP, SBP, PP, and HR were similar in the two groups, but resting DBP was higher in the older group than the young group (*P *< 0.05). FO significantly increased EPA and DHA levels in both age groups. This increase was greater in the older compared to the young group for EPA (*P *< 0.05), and tended to be greater in the older compared to the young group for DHA (*P* = 0.078). Due to these effects, EPA was higher (*P *< 0.05) and DHA tended to be higher (*P* = 0.056) in the older compared to the young group. FO did not affect baseline cardiovascular values between visits.

**Table 1:** Baseline cardiovascular, and EPA and DHA content, values of the young and older groups

	Young		Older	
	Pre‐FO	Post‐FO	Pre‐FO	Post‐FO
MAP (mmHg)	86 ± 1	86 ± 1	89 ± 2	88 ± 3
SBP (mmHg)	117 ± 3	117 ± 3	120 ± 3	121 ± 3
DBP (mmHg)	67 ± 1	67 ± 1	72 ± 2^a^	72 ± 2^a^
PP (mmHg)	50 ± 2	50 ± 3	48 ± 2	48 ± 3
HR (b.min^−1^)	60 ± 3	58 ± 3	62 ± 2	60 ± 2
Erythrocyte EPA content (%)	0.52 ± 0.04	1.83 ± 0.38^b^	0.71 ± 0.09^a^	3.21 ± 0.28^a^^,^ b ^,^ c
Erythrocyte DHA content (%)	4.17 ± 0.22	6.21 ± 0.50^b^	4.44 ± 0.45	7.74 ± 0.30^b^

Data are shown as means ± SEM.

DBP, diastolic blood pressure; DHA, docosahexaenoic acid; EPA, eicosapentaenoic acid; FO, fish oil; HR, heart rate; MAP, mean arterial blood pressure; PP, pulse pressure; SBP, systolic blood pressure.

a Significantly different from young (*P* < 0.05).

b Significant effect of FO (*P *< 0.05).

c Significantly different from young post‐FO (*P *< 0.05).

### Handgrip exercise

Maximal voluntary contraction values were similar in the two age groups (young pre‐FO: 39.6 ± 1.7 kg; older pre‐FO: 34.2 ± 3.1 kg). Handgrip force was significantly greater with increasing exercise intensity in both age groups (young pre‐FO: 4.0 ± 0.2 kg at 10%, 10.9 ± 0.5 kg at 30%, 18.9 ± 0.9 kg at 50%, and 25.0 ± 1.1 kg at 70% MVC; older pre‐FO: 3.7 ± 0.3 kg at 10%, 9.7 ± 0.9 kg at 30%, 16.2 ± 1.5 kg at 50%, and 22.8 ± 2.1 kg at 70% MVC). FO did not affect MVC (young post‐FO: 39.7 ± 2.2 kg; older post‐FO: 34.8 ± 3.2 kg) or handgrip force values (young post‐FO: 4.2 ± 0.3 kg at 10%, 11.4 ± 0.7 kg at 30%, 19.2 ± 1.0 kg at 50%, and 26.6 ± 1.4 kg at 70% MVC; older post‐FO: 3.6 ± 0.3 kg at 10%, 10.0 ± 0.9 kg at 30%, 16.7 ± 1.5 kg at 50%, and 22.9 ± 2.0 kg at 70% MVC) between visits.

### Cardiovascular responses to handgrip pre‐ and post‐FO

**Figure 2:**
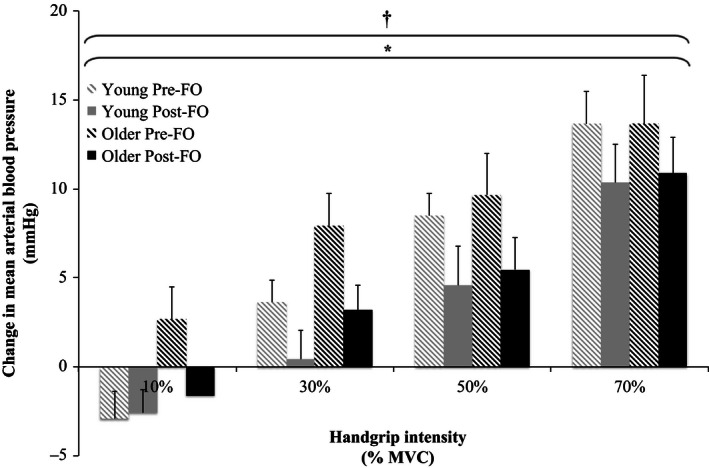
Relative changes from baseline in mean arterial blood pressure in response to 10%, 30%, 50%, and 70% maximum voluntary contraction (MVC) handgrip exercise in the young and older groups pre‐ and post‐fish oil (FO). ^†^Significant effect of handgrip (*P *< 0.05). *Significant effect of FO (*P *< 0.05).

**Figure 3:**
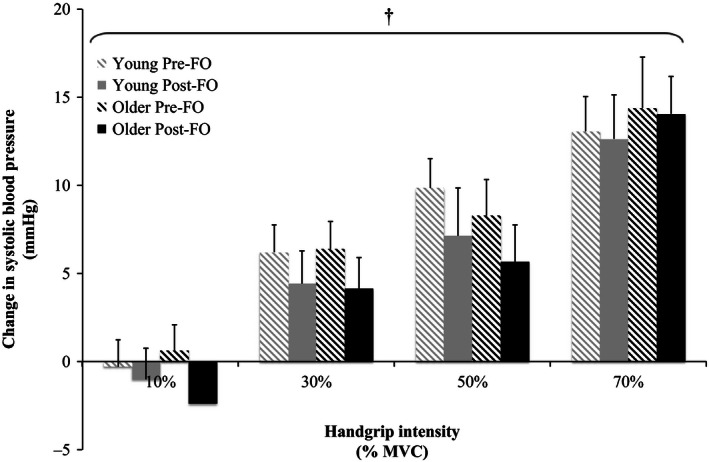
Relative changes from baseline in systolic blood pressure in response to 10%, 30%, 50%, and 70% maximum voluntary contraction (MVC) handgrip exercise in the young and older groups pre‐ and post‐fish oil (FO). ^†^Significant effect of handgrip (*P *< 0.05).

**Figure 4:**
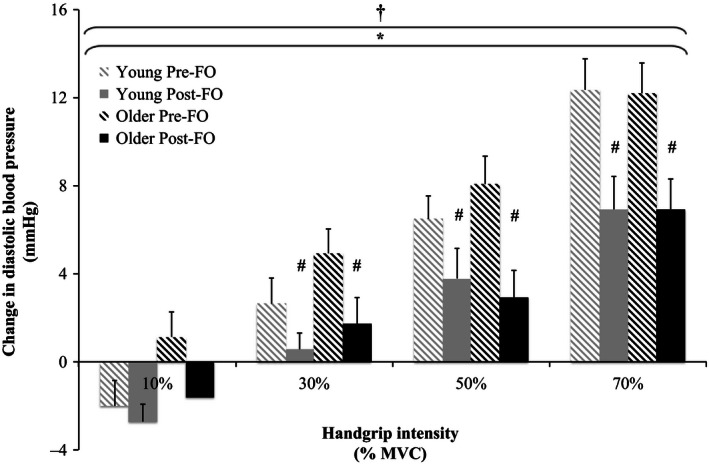
Relative changes from baseline in diastolic blood pressure in response to 10%, 30%, 50%, and 70% maximum voluntary contraction (MVC) handgrip in the young and older groups pre‐ and post‐fish oil (FO). ^†^Significant effect of handgrip (*P *< 0.05). *Significant effect of FO (*P *< 0.05). ^#^Significantly different from pre‐FO (*P *< 0.05).

**Figure 5:**
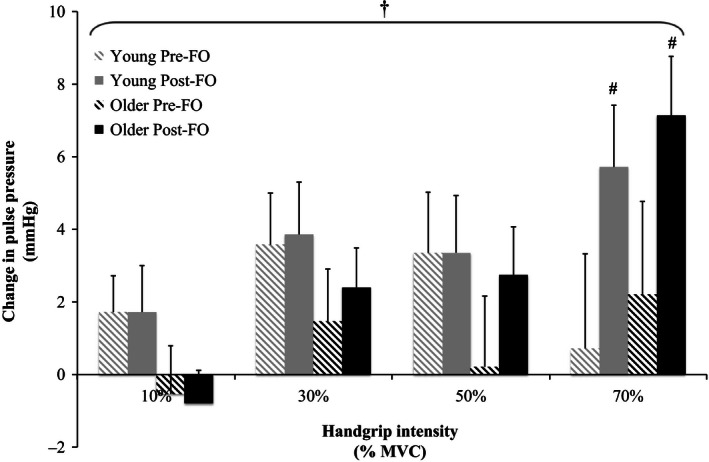
Relative changes from baseline in pulse pressure in response to 10%, 30%, 50%, and 70% maximum voluntary contraction (MVC) handgrip in the young and older groups pre‐ and post‐fish oil (FO). ^†^Significant effect of handgrip (*P *< 0.05). ^#^Significantly different from pre‐FO (*P *< 0.05).

**Figure 6:**
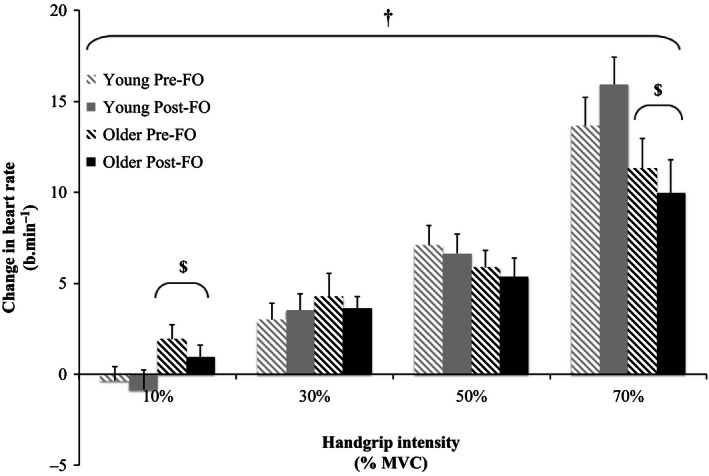
Relative changes from baseline in heart rate in response to 10%, 30%, 50%, and 70% maximum voluntary contraction (MVC) handgrip in the young and older groups pre‐ and post‐fish oil (FO). ^†^Significant effect of handgrip (*P *< 0.05). ^$^Significantly different from young (*P *< 0.05).

Relative changes from baseline in MAP, SBP, DBP, PP, and HR in response to the graded isometric handgrip trials pre‐ and post‐FO are shown in Figures 2, 3, 4, 5, 6. MAP, SBP, DBP, PP, and HR all increased in response to isometric handgrip exercise in both the young and older groups, with progressively greater increases in response to higher intensities of handgrip (*P *< 0.05). FO supplementation attenuated the increases in MAP (Fig. 2) and DBP (Fig. 4) in response to handgrip in both age groups (*P *< 0.05). Specifically, FO supplementation reduced the increases in DBP in response to handgrip of higher intensities (*P *< 0.05; Fig. 4). FO supplementation did not affect SBP increases in response to handgrip in either age group (Fig. 3). At the highest intensity of handgrip (70% MVC), FO supplementation augmented the PP increase in both age groups (*P *< 0.05). The older group had greater HR increases in response to the lowest intensity of handgrip (10% MVC), and blunted HR increases at the highest intensity of handgrip (70% MVC) compared to the young group (*P *< 0.05). FO supplementation did not affect HR increases in response to handgrip in either age group.

## Discussion

The main finding from this study is that daily FO supplementation for 12 weeks attenuated increases in MAP and DBP at the onset of isometric handgrip exercise in healthy young and older subjects. This finding suggests that dietary intake of omega‐3 polyunsaturated fatty acids reduces the cardiovascular response to acute physiological stress, and this modulatory effect occurs irrespective of age.

Previous studies have revealed that older individuals exhibit smaller increases in HR and cardiac output, but a similar MAP increase, at the onset of isometric handgrip exercise compared to young individuals (Momen et al. 2004; Lalande et al. 2014). This attenuated HR increase at the onset of exercise in older people has been attributed to the overall reduction in parasympathetic nerve activity, and therefore level of parasympathetic withdrawal that occurs at this time of rapid reflex neural adjustments (Stratton et al. 2003), and/or reduced cardiac M_2_ muscarinic receptor density and function (Poller et al. 1997; Brodde et al. 1998), both of which manifest with advancing age. The preserved MAP increase is likely a consequence of increased arterial stiffness with age, which offsets the reduced cardiac output increase at the onset of exercise (Momen et al. 2004; Lalande et al. 2014). Our results are concordant with these previous findings, as we also observed that older subjects had a smaller increase in HR at the onset of the highest intensity of isometric handgrip exercise compared to the young subjects. Additionally, both age groups exhibited similar increases in MAP, SBP, DBP, and PP at exercise onset across all intensities of isometric handgrip performed.

We observed that daily intake of FO for 12 weeks attenuated increases in both MAP and DBP at the onset of isometric handgrip exercise in both age groups. The smaller DBP increase with FO supplementation was likely the driving factor for the reduced MAP increase with FO supplementation that we observed, due to the greater relative time in diastole than systole in the cardiac cycle and the resulting larger influence of DBP on MAP. Further, our finding that the PP increase at the onset of the highest intensity of isometric handgrip exercise was augmented in both age groups following FO supplementation reflects the attenuated DBP increase yet unaffected SBP increase, resulting in a widened PP, at this time.

Although the objective of this study was not to identify the specific mechanism(s) underlying the effect of FO intake on the MAP response at the onset of isometric handgrip exercise in young and older subjects, one possible explanation for our findings is related to nitric oxide production. Previous studies in animals have demonstrated that FO intake enhances the generation and bioavailability of nitric oxide, an endogenous vasodilator, and augments the production of endothelial nitric oxide synthase, the enzyme responsible for the synthesis of nitric oxide (Nishimura et al. 2000; Lopez et al. 2004). Studies in humans have shown that FO intake increases nitric oxide production or release, and results in greater endothelium‐dependent vasodilation in healthy subjects (Walser et al. 2006), and patients with coronary artery disease (Tagawa et al. 1999) and type 2 diabetes mellitus (McVeigh et al. 1993), with both of these patient groups likely exhibiting endothelial dysfunction. Thus, it is possible that FO supplementation could have resulted in greater nitric oxide production, thereby leading to the attenuated DBP and therefore MAP increases in response to handgrip exercise via vasodilatory effects on the endothelium. Investigations specifically addressing this concept are warranted, particularly due to the potential cardiovascular benefit that FO supplementation may provide.

We hypothesized that older subjects would exhibit an attenuated MAP increase at the onset of isometric handgrip exercise following 12 weeks of FO supplementation, which our findings support, but we had not anticipated that this modulatory effect of FO supplementation would also occur in young subjects. As older individuals exhibit a preserved increase in MAP at the onset of isometric handgrip exercise, likely due to greater arterial stiffness with advancing age offsetting the smaller cardiac output increase at the onset of handgrip (Momen et al. 2004; Lalande et al. 2014), our recent finding that FO supplementation decreases central arterial stiffness in older but not young subjects (Monahan et al. 2015) led us to hypothesize that FO supplementation would attenuate the MAP increase at the onset of isometric handgrip in older subjects by decreasing their central arterial stiffness. However, as FO supplementation did not alter central arterial stiffness in young individuals in our recent study (Monahan et al. 2015), it is not clear whether FO‐related alterations in central arterial stiffness are responsible for mediating the attenuated MAP and DBP increases in response to isometric handgrip in this investigation.

This study has several limitations. First, a placebo group was not included as part of the study design. Second, EPA and DHA were quantified in only a subset of subjects; however, the data included from nine young and eight older subjects likely provide a good indication of the effect of FO supplementation on these omega‐3 polyunsaturated fatty acid levels. Third, we found that EPA and DHA levels were greater in the older as compared to the young group following FO supplementation. Our previous study reported similar findings (Monahan et al. 2015); however, we are unaware of existing literature that could explain the disparate responses to FO supplementation in the two age groups in this study. Given the potential health benefits associated with FO supplementation, this could be an important area in which further study is warranted.

### Perspectives and significance

Our finding that 12 weeks of FO supplementation attenuated the MAP and DBP increases at the onset of isometric handgrip exercise in healthy young and older subjects provides novel insight into the potential cardiovascular health benefits of omega‐3 polyunsaturated fatty acid consumption. Lowering cardiovascular responses to acute physiological stress in older people has clinical relevance, as this population is at increased risk of suffering a cardiac or cerebral event, as well as developing cardiovascular disease. The physiological stress of brief isometric handgrip exercise used in this study mimics some actions involved in daily living, such as holding or carrying items with one or both hands for a short period of time, and so these findings could have implications for everyday activities. This possible cardioprotective effect of FO supplementation at a time of acute physiological stress may be another way in which omega‐3 polyunsaturated fatty acid consumption reduces cardiovascular risk, in addition to the known beneficial effects of omega‐3 polyunsaturated fatty acid consumption. The effect of FO supplementation of reducing the rise in MAP and DBP at the onset of isometric handgrip exercise occurred in subjects of good cardiovascular health, including healthy older subjects, and so it is possible that this potentially beneficial effect of FO supplementation could be of even further benefit to people with poorer cardiovascular health or cardiovascular disease. With the relative proportion of older people in the population increasing, a greater number of people have elevated cardiovascular risk, and therefore further investigations in this clinically significant area of potential therapeutic benefit with FO supplementation are needed.

## Conflict of Interest

There are no conflicts of interest.
